# Engineering a Modular PapMV Nanoparticle Vaccine: Comparative Efficacy of a Covalent and a Non-Covalent N-Antigen Vaccine Against Emerging SARS-CoV-2 Variants

**DOI:** 10.3390/vaccines14040349

**Published:** 2026-04-15

**Authors:** Léa-J. Blanchette, Marilène Bolduc, Tekeleselassie Woldemariam, Mitra Yousefi, Henintsoa Rabezanahary, Santa-M. Olivera-Ugarte, Caroline Garneau, Myriam Angers, Rong Shi, Louis Flamand, Mariana Baz, Silvia Vidal, Darryl Falzarano, Jean-François Lemay, Denis Leclerc

**Affiliations:** 1 Centre de Recherche du CHU de Québec, Université Laval, Axe Immunology and Infectious Diseases, 2705 Boul Laurier, Québec City, PQ G1V 4G2, Canadamarilene.bolduc@crchudequebec.ulaval.ca (M.B.);; 2 Vacine and Infectious Disease Organization (VIDO), Department of Veterinary Microbiology, University of Saskatchewan, 120 Veterinary Road, Saskatoon, SK S7N 5E3, Canada; 3 McGill Life Sciences Complex, Bellini Building, 3649 Promenade Sir-William-Osler, Montreal, PQ H3G 0B1, Canada; mitra.yousefi@mcgill.ca (M.Y.);; 4 Institut de Biologie Intégrative et des Systèmes, Pavillon Eugène-Marchand, Université Laval, 1030 Avenue de la Médecine, Québec City, PQ G1V 0A6, Canada; 5 Centre National en Électrochimie et en Technologies Environnementales Inc., 2263 Av. du Collège Porte 7, Shawinigan, PQ G9N 6V8, Canada

**Keywords:** nanoparticle, SARS-CoV-2, vaccine, Papaya Mosaic Virus (PapMV), TLR7/8 agonist

## Abstract

**Background**: Despite the effectiveness of current SARS-CoV-2 vaccines, the genetic variability of the viral target has led to the emergence of variants capable of evading vaccine-induced protection. To ensure broader and more durable protection, we investigated the efficacy of a novel vaccine strategy. **Methods**: This vaccine utilizes the highly conserved nucleocapsid (N) protein as its primary antigen, rather than the spike (S) protein. It incorporates the Papaya Mosaic Virus (PapMV) nanoparticle, a Toll-like receptor (TLR) 7/8 agonist with intrinsic adjuvant properties, as a vaccine platform. **Results**: The vaccine formulations, comprising PapMV nanoparticles and the N antigen covalently or non-covalently attached to the PpaMV nano, generated robust humoral (antibody) and cellular (T-cell) immune responses. Protective efficacy was evaluated in K18-hACE2 transgenic mice challenged with either the ancestral SARS-CoV-2 strain or the Omicron XBB.1.5 variant. In both cases, the vaccine significantly reduced inflammation and viral titers in the lungs of vaccinated animals. **Conclusions**: These results highlight the potential of this PapMV-N vaccine to induce broad protection against diverse SARS-CoV-2 variants.

## 1. Introduction

Despite the efficacy of mRNA vaccines, new SARS-CoV-2 variants have emerged with increased transmissibility and the ability to evade vaccine-induced protection [1,2,3,4]. Numerous studies have identified a high frequency of amino acid substitutions in the Spike (S) protein, particularly within the receptor-binding domain (RBD), leading to significant humoral immune evasion in vaccinated or previously infected individuals [1,2,3,5]. Furthermore, repeated administration of S-protein-based vaccines has resulted in immune imprinting, potentially constraining long-term vaccine effectiveness [6,7].

To enhance efficacy and elicit broader immunity, the use of the SARS-CoV-2 nucleocapsid (N) protein as a vaccine antigen has garnered significant interest [8,9]. The N protein is expressed at high levels during viral replication [10,11], and its sequence remains highly conserved across variants [8,9]. For instance, a comparison between the ancestral strain and the XBB.1.5 variant, representing opposite ends of the SARS-CoV-2 evolutionary spectrum, reveals 99% identity in the N protein. Importantly, this conserved protein harbors several T-cell epitopes that have been shown to elicit broad protection [8,9,12,13,14].

In this report, we investigated a novel nanoparticle vaccine platform combined with the SARS-CoV-2 N protein. We evaluated the effectiveness of this formulation in inducing a protective immune response against diverse SARS-CoV-2 variants. The nanoparticle, derived from the self-assembly of the Papaya Mosaic Virus (PapMV) coat protein and a 1517-nucleotide single-stranded RNA (ssRNA), possesses several features that make it an ideal vaccine platform.

First, the PapMV nanoparticle (PapMV-nano) elicits a strong Th1-biased immune response toward conjugated antigens, characterized by the production of IgG2a (Balb/C) or IgG2c (C57BL/6) antibodies in mice, which bind with higher avidity and trigger antibody-dependent cell-mediated cytotoxicity (ADCC) more effectively than other subclasses [15,16,17,18,19]. Second, unlike other protein-based platforms such as hepatitis B virus (HBV) or ferritin-based nanoparticles, PapMV-nano uniquely triggers the innate immune system via Toll-like receptors (TLR) 7 and 8, leading to IFN-α secretion [17,20,21]. This inherent adjuvant activity broadens the subsequent immune response [15,16,17,18,19,21]. Third, the platform is highly versatile; it utilizes the bacterial transpeptidase Sortase A (SrtA) to covalently attach full-length protein antigens to its surface, thereby enhancing antigenic presentation to B cells and boosting the humoral response [17,18,19]. Fourth, production in standard recombinant bacterial systems facilitates rapid scale-up and lower manufacturing costs compared to live-attenuated or viral-vector vaccines [15,16,17,18,19,21]. Finally, the PapMV platform has been shown to be safe in humans and carries no risk of genomic integration [22].

In this study, we developed two vaccine formulations, comprising PapMV-nano and the N antigen. In the first formulation, the N antigen was covalently attached to the surface of the PapMV nano while, in the second formulation, a non-covalent complex between the PapMV nano, the SrtA and the N protein was generated. We evaluated their immunogenicities and protective capacities against both the ancestral SARS-CoV-2 strain and the XBB.1.5 variant in a mouse model. 

## 2. Materials and Methods

### 2.1. Production and Purification of Recombinant Proteins

*LPS removal:* In all cases, the purified proteins were filtered through a Sartobind Q (Sartorius, Göttingen, Germany) to remove contaminating lipopolysaccharides (LPSs). Final LPS levels remained below 5 EU/mg of protein in all preparations, as assessed using an Endosafe Nexgen-PTS system (Charles River, Wilmington, MA, USA).

*PapMV nanoparticles:* The design, production, and purification of the PapMV nanoparticles were performed as previously described [18]. Briefly, the recombinant PapMV coat protein (CP), containing four glycines at the N-terminus, was fused N-terminally to an intein tag. This chimeric protein (Intein-PapMV-CP), which also included a C-terminal 6xHis-tag, was expressed in *E. coli* (strain BD-792) and purified via immobilized metal affinity chromatography (IMAC). Cleavage of the intein was induced by incubation at pH 6.5, and the PapMV CP was subsequently recovered by IMAC to achieve >95% purity. Finally, the PapMV nanoparticles were assembled in vitro using a synthetic non-coding single-stranded RNA (ssRNA) derived from the native PapMV viral genome. The molecular weight of the PapMV monomers is approximately 24.15 kDa, and the resulting nanoparticles range from 65 to 100 nm in size. Batches of PapMV nanoparticles were stored in 10 mM Tris-HCl (pH 8.0).

*Sortase A (SrtA):* The production and purification of the transpeptidase SrtA were performed as previously described [19]. Briefly, N-terminally 6xHis-tagged SrtA was produced in *E. coli* (strain BD-792) and purified by IMAC to achieve > 95% purity. The molecular weight of SrtA is 21.69 kDa. Purified batches were stored in 50 mM Tris-HCl and 150 mM NaCl (pH 7.5).

*Nucleocapsid (N):* The recombinant SARS-CoV-2 N protein from the ancestral strain (Wuhan-1) was cloned into the pQE-80L plasmid. Expression was conducted in *E. coli* strain BD-792 under the control of the T5 promoter (Qiagen, Hilden, Germany). The N protein construct includes the SrtA recognition motif (LPETGG) followed by a C-terminal 6xHis tag. Cultures were grown at 37 °C in 1 L of 2xYT medium supplemented with 25 µg/mL kanamycin until reaching an OD_600_ of 1.0. Protein expression was induced with 1 mM isopropyl-β-D-thiogalactopyranoside (IPTG) (Millipore, Darmstadt, Germany) for 7 h at 20 °C.

Cells were harvested by centrifugation (5000× *g*, 5 min, 4 °C) and resuspended in 100 mL of ice-cold lysis buffer (10 mM Tris-HCl pH 8.0, 20 mM NaCl, and 10 mM imidazole). Lysozyme (0.3 mg/mL) and PMSF (1 mM) were added, followed by a 30 min incubation at 4 °C. The bacteria were lysed using two passes through an Emulsiflex C5 homogenizer (Avestin, Ottawa, ON, Canada) at 18,000–22,000 psi. Benzonase (2500 units; Sigma-Aldrich, St. Louis, MO, USA) and 2 mM MgCl_2_ were added to the lysate, incubated for 30 min at room temperature, and clarified by centrifugation (15,000× *g*, 15 min).

The clarified supernatant was loaded onto a 15 mL Ni-NTA agarose column (Qiagen, Toronto, ON, Canada) at 4 °C. The column was washed sequentially with 10 column volumes (CVs) of Wash Buffer 1 (10 mM Tris–HCl pH 8.0, 100 mM NaCl, 10 mM imidazole), Wash Buffer 2 (25 mM imidazole), and Wash Buffer 3 (40 mM imidazole). The N protein was eluted with 1 CV of Elution Buffer (500 mM imidazole). Finally, the protein was dialyzed against PBS (137 mM NaCl, 2.7 mM KCl, 10 mM Na_2_HPO_4_, 1.8 mM KH_2_PO_4_, pH 7.4), filtered (0.2 µm), and stored at −80 °C.

### 2.2. Coupling Reaction Using SrtA Transpeptidase

The conjugation of recombinant proteins to PapMV nanoparticles was performed as previously described [23,24,25]. Briefly, the N protein was coupled to PapMV nanoparticles by incubation at 21 ± 1 °C with SrtA in a transpeptidation buffer (50 mM Tris-HCl, 150 mM NaCl, 10 mM CaCl_2_; pH 7.5). Coupling conditions were optimized by systematically varying the incubation time, protein ratios, and SrtA concentration. Reaction turbidity was monitored throughout the process. Upon completion, SrtA activity was quenched by adding ethylenediaminetetraacetic acid (EDTA) to chelate Ca^2+^ and terminate the reaction.

### 2.3. Purification and Formulation Optimization

Several purification strategies for PapMV-N were evaluated. Different dialysis membranes (100 kDa Biotech Cellulose Ester [CE] and 300 kDa Float-A-Lyzer G2, (Repligen Corporation, Waltham, MA, USA) were tested to assess the removal of SrtA and uncoupled N protein. Centrifugal filtration was also evaluated using Amicon^®^ Ultra 100 kDa devices (Millipore, Burlington, MA, USA). Additionally, size-exclusion chromatography (Superdex 200, Cytiva, Marlborough, MA, USA) and IMAC were tested for PapMV-N purification. To prevent protein aggregation, various formulation trials were conducted. PapMV-N was buffer-exchanged into 10 mM Tris-HCl (pH 8.0) or 150 mM NaCl with 10 mM CaCl_2_ (pH 7.5). Stabilizing additives, including crowding agents, were tested to maintain the stability of the coupled complex. Protein turbidity was assessed by measuring the OD_600_ using a DeNovix DS-11 Series spectrophotometer (Wilmington, DE, USA), as previously described [26].

### 2.4. Physico-Chemical Characterization of the PapMV-N Vaccine and Its Components

*SDS-PAGE and Immunoblotting:* PapMV-N coupling reactions were diluted with 5X loading buffer containing 2-mercaptoethanol and bromophenol blue, then incubated at 95 °C for 10 min to reduce the proteins to their monomeric state. Samples were loaded onto 10% Tris-Glycine gels for SDS-PAGE. Gels were either stained with GelCode Blue Stain Reagent (Thermo Fisher, Waltham, MA, USA) or transferred onto a PVDF membrane for Western blot analysis. Membranes were blocked with 5% skimmed milk powder and incubated for 18 ± 2 h with either a rabbit anti-PapMV polyclonal antibody (1/10,000 in 5% milk) or a rabbit anti-SARS-CoV-2 nucleocapsid (N) polyclonal antibody (1/2000 in 5% milk). After washing with Tris-buffered saline (TBS) containing Tween-20, membranes were incubated for two hours with a goat anti-rabbit IgG conjugated to alkaline phosphatase (1/25,000 in 5% milk). Protein bands were visualized using the BCIP^®^/NBT-Blue Liquid Substrate System (Millipore Sigma, Burlington, MA, USA).

*Assessment of coupling efficiency:* Coupling efficiency was analyzed using ImageJ software (version 1.49). The percentage of conjugation was determined by measuring the intensity of the coupled band relative to the total intensity of both coupled and uncoupled PapMV CP. The coupled band (PapMV CP linked to the N protein) has a theoretical molecular weight (MW) of approximately 71.2 kDa (PapMV CP: 24.2 kDa; N protein: 47 kDa). Efficiency was calculated as follows:

Coupling Efficiency (%) = [AUC of coupled PapMV CP/(AUC of coupled PapMV CP + AUC of uncoupled PapMV CP)] × 100.

*Dynamic Light Scattering (DLS):* The size distribution of PapMV nanoparticles was estimated by DLS using a ZetaSizer Nano ZS (Malvern, UK). Samples were diluted to 0.25 mg/mL, as determined by bicinchonic acid assay protein assay (BCA) (Thermo Fisher, Waltham, MA, USA), in 10 mM Tris-HCl (pH 8.0) and measured in disposable plastic cuvettes.

*Transmission Electron Microscopy (TEM):* The morphology of the coupled nanoparticles was examined using an FEI Tecnai Spirit transmission electron microscope (FEI, Hillsboro, OR, USA). Samples were diluted to 0.02 mg/mL, mixed 1:1 with a 3% uranyl acetate solution, and incubated for 7 min in the dark. The mixture was then applied to carbon/Formvar-coated grids for 5 min. Excess solution was removed, and grids were air-dried for 2–3 h prior to imaging. 

### 2.5. Vaccine Formulation and Preparation

*CpG oligonucleotide + N protein (CpG + N):* ODN 2395 (Class C CpG ODN; InvivoGen, San Diego, CA, USA, Cat. #tlrl-2395) was used throughout the animal experiments. According to the manufacturer’s guidelines, each vaccine dose consisted of 50 µg of CpG adjuvant combined with 9 µg of N protein in a formulation buffer of 10 mM Tris-HCl and 150 mM NaCl (pH 8.0).

*Aluminum phosphate + N protein (Alum + N):* The aluminum phosphate adjuvant Adju-Phos^®^ (InvivoGen, San Diego, CA, USA, Cat. #vac-phos-250) was utilized. Adju-Phos^®^ carries a negative charge at pH 5–7, making it compatible with the positively charged N protein (isoelectric point: 10.5). This facilitates antigen adsorption onto the aluminum salt particles, enhancing recognition and internalization by immune cells. Following the manufacturer’s recommended volume ratio (1:9 to 1:1, Adju-Phos^®^:antigen), each 100 µL dose was prepared by combining 50 µL of Alum with 9 µg of N protein in 10 mM Tris-HCl and 150 mM NaCl (pH 8.0).

*PapMV-N and PapMV + N vaccines:* Based on optimization trials, the optimal protein ratio for the PapMV-N vaccine candidate was determined to be 2 µM N protein/25 µM SrtA/50 µM PapMV nanoparticles. For a 100 µL dose, this corresponds to 9 µg of N protein, 53 µg of SrtA, and 118 µg of PapMV nanoparticles in a formulation buffer containing 50 mM Tris-HCl, 150 mM NaCl, 10 mM CaCl_2_, and 10 mM EDTA (pH 7.5). For the uncoupled PapMV + N control, the same protein quantities were mixed immediately prior to immunization to prevent conjugation. 

### 2.6. Mouse Immunizations

Female C57BL/6 mice (6–8 weeks old; Jackson Laboratory, Bar Harbor, ME, USA) were used to evaluate the immunogenicity of the vaccine candidates. Groups of 5 or 10 mice, as specified in the figure legends, were immunized intramuscularly with 100 µL of the respective vaccine candidates (PapMV-N, PapMV + N, CpG + N, or Alum + N) or control formulations (PapMV or formulation buffer), with 50 µL administered per hamstring. Immunizations were performed on days 0 and 21. Blood samples were collected via submandibular bleeding prior to immunization (naïve sera) and on days 20 and 35 to measure anti-N antibody titers. On day 35, mice were euthanized, and spleens were harvested for IFN-γ ELISpot analysis.

### 2.7. Antibody Titration by ELISA

As previously described [18], anti-N IgG2c and IgG1 antibodies were measured in the sera of C57BL/6 mice after the first (day 20) and second (day 35) immunizations by enzyme-linked immunosorbent assay (ELISA). Results are reported as endpoint titers, defined as the highest dilution exceeding three times the OD_450nm_ value of the background (measured using a pool of pre-immune sera).

### 2.8. IFN-γ ELISpot Assay

An enzyme-linked immunospot (ELISpot) assay was performed to quantify the frequency of interferon-gamma (IFN-γ) secreting cells in the spleens of immunized C57BL/6 mice using a murine IFN-γ ELISpot Kit (Abcam, Cambridge, UK) according to the manufacturer’s instructions. Briefly, MultiScreen-IP filter plates (MilliporeSigma, Burlington, MA, USA) were coated with an IFN-γ capture antibody for 18 ± 2 h at 4 °C. Splenocytes were isolated, counted using an Invitrogen™ Countess™ 3 FL automated cell counter (Waltham, MA, USA), and seeded at 5 × 10^5^ cells per well. Cells were stimulated with either the N protein (50 µg/mL), Concanavalin A (5 µg/mL; positive control), or culture medium (negative control). After incubation for 20 h at 37 °C with 5% CO_2_, the plates were processed and spots were quantified using an ImmunoSpot S6 reader (C.T.L.) (Shaker Heights, OH, USA).

### 2.9. Microscale Thermophoresis (MST) Binding Studies

MST experiments were conducted using a Monolith NT.115 instrument (NanoTemper Technologies GmbH, Munich, Germany) at 22 °C in transpeptidation buffer (50 mM Tris-HCl, 150 mM NaCl, 10 mM CaCl_2_, pH 7.5). PapMV nanoparticles were labeled with Cy5 fluorescent dye (approximately one fluorophore per five monomeric units). Interactions between the labeled PapMV nanoparticles and unlabeled ligands induce perturbations in fluorescence emission, recorded at 650/670 nm. Dissociation constants (*Kd*) were determined by plotting the fluorescence intensity ratios against the ligand concentrations and calculated using GraphPad Prism 9.0 (GraphPad Software Inc., La Jolla, CA, USA).

To evaluate binding affinities, the concentration of Cy5-labeled PapMV nanoparticles was kept constant (0.698 µM) while the unlabeled partners were serially diluted (1:2). The ligands tested included the N protein (concentration range: 92.25 µM to 0.14 nM), SrtA (272.7 µM to 0.41 nM), and a monoclonal antibody directed against the PapMV surface (positive control; 3.335 µM to 0.019 nM). To assess tripartite interactions, the *Kd* was determined by keeping two components at constant concentrations—either Cy5-PapMV (0.698 µM) and SrtA (272.7 µM) while varying the N protein, or Cy5-PapMV (0.698 µM) and N protein (92.25 µM) while varying SrtA. All conditions were performed in triplicate.

### 2.10. K18-hACE2 Mouse Viral Challenge

Female K18-hACE2 (Bar Harbor, ME, USA) transgenic mice (6–8 weeks old; Jackson Laboratory, USA) were used for the infectious challenge. Ten mice per group were immunized according to the schedule described above. Five mice received formulation buffer to serve as non-infected (mock) controls. At day 42, mice were challenged intranasally with $10^{3}$ TCID_50_ (50% tissue culture infectious dose) of the SARS-CoV-2 ancestral strain (Wuhan-1), provided by the *Institut national de santé publique du Québec* (INSPQ). Body weight, survival, and clinical symptoms were monitored daily for 7 days post-infection (p.i.) using a blinded procedure. On day 5 p.i., five mice per group were euthanized; nasal turbinates and lungs were harvested for viral titration and cytokine analysis of lung homogenate supernatants. On day 7 p.i., the remaining mice (*n* = 5) and the mock group were sacrificed to harvest nasal turbinates, lungs, and brain for viral titration. Lung histopathology was also performed on three mice per group on day 7 p.i.

### 2.11. Lung Histopathology

Lungs collected at day 7 p.i. were fixed in 10% buffered formalin, embedded in paraffin, and sectioned at 4 μm. Sections were stained with hematoxylin and eosin (H&E). Histopathological inflammatory scores (ISs) were assessed by an experienced pathologist using a semi-quantitative scale covering bronchial, peribronchial, perivascular, interstitial, pleural, and intra-alveolar regions. Capillary congestion, edema, and the nature of the inflammatory infiltrate (acute/neutrophilic vs. chronic/lymphohistiocytic) were also evaluated. Results are expressed as total pulmonary inflammatory scores (TISs).

### 2.12. Viral Titer Assessment

Lung tissues were weighed and homogenized using a TissueLyser II (Qiagen, Hilden, Germany) at 30 Hz for 6 min in DMEM (Sigma, St. Louis, MO, USA) with a stainless-steel bead. Homogenates were clarified by centrifugation (5000× *g*, 5 min). Ten-fold serial dilutions of the supernatant were prepared in 96-well plates. Vero 76 cells (ATCC CRL-1587) were inoculated with each dilution in triplicate and incubated for 1 h at 37 °C (5% CO_2_). The inoculum was then replaced with DMEM supplemented with 2% FBS and 1% Pen-Strep. Cells were monitored for cytopathic effect (CPE) on days 1, 3, and 5. The TCID_50_ was calculated using the Spearman–Kärber method [27,28].

### 2.13. RT-qPCR for Lung IL-6 Expression

Total RNA was extracted from weighed lung tissue homogenized in RLT buffer (Qiagen) containing 1% β-mercaptoethanol. Viral RNA was purified using the RNeasy Mini Kit (Qiagen) according to the manufacturer’s instructions. RT-qPCR was performed on a QuantStudio 3 system (Thermo Fisher Scientific, Waltham, MA, USA) using the One-Step TB Green PrimeScript RT-PCR Kit II (Takara, San Jose, CA, USA). Cycling conditions were: 42 °C for 5 min (RT), 95 °C for 10 s (initial denaturation), followed by 40 cycles of 95 °C for 5 s and 60 °C for 30 s. IL-6 expression was normalized to the GAPDH housekeeping gene.

### 2.14. Statistical Analysis

Statistical analysis was performed using GraphPad Prism 7.0b. Differences in antibody titers, ELISpot counts, and viral titers were analyzed by one-way ANOVA followed by Tukey’s post hoc test. Two-way ANOVA with Tukey’s test was used for cytokine concentrations and histopathological scores. A *p*-value < 0.05 was considered statistically significant. 

## 3. Results

### 3.1. Production of Recombinant Proteins

**Figure A1:**
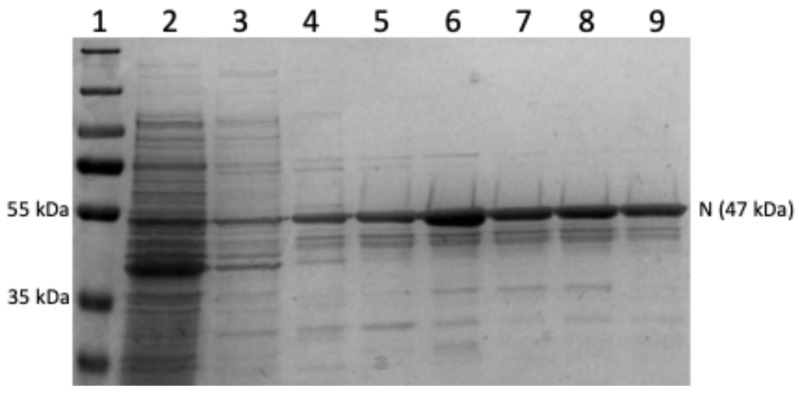
SDS-PAGE showing the purification of the SARS-CoV-2 N protein from bacterial lysate. The N protein from the ancestral variant of SARS-CoV-2 was cloned in the expression vector pQE80 used to transform the *E. coli* strain BD-792. Upon expression, the N protein, found at 47 kDa, accumulated at high levels in bacteria (see lane 2). The N protein, containing the SrtA coupling signal followed by a 6xH tag, was purified by ion metal affinity chromatography (IMAC) to yield a N protein of 47 kDa that is estimated at 95% of purity (see lane- purified). Lane 1: MW markers; lane 2: flow through of the lysate after passage on the IMAC column; lane 3: first wash; lane 4: second wash; lane 5: third wash; lane 6: elution of the N protein using imidazole; lane 7: elution after dialysis to remove imidazole; lane 8: elution after dialysis and filtration through a 0.45 µM filter; lane 9: elution after dialysis, filtration through a 0.45 µM and a 0.2 µM filter. This protein was stored at −80 °C.

The SARS-CoV-2 N protein from the ancestral variant was cloned, expressed in *E. coli*, and purified via immobilized-metal affinity chromatography (IMAC) (Figure A1). Production and purification of the PapMV nano and SrtA were performed as described [18].

### 3.2. Conjugation of the SARS-CoV-2 N Antigen to the PapMV Nanoparticle Surface

**Figure 1:**
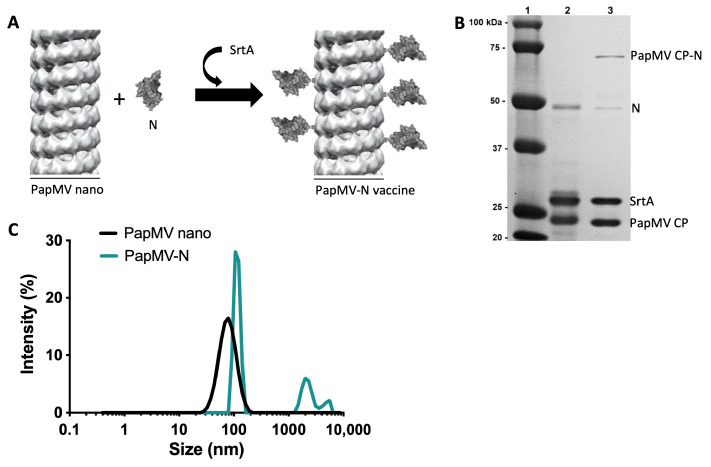
Coupling reaction between the SARS-CoV-2 N protein and the PapMV nano. (**A**) Schematic representation of the coupling reaction created with BioRender (BioRender.com). The PapMV nano is mixed with the recombinant N protein and the SrtA in a coupling reaction leading to the generation of the PapMV-N. (**B**). SDS-PAGE analysis of the coupling reaction on a Tris-Glycine 10% gel. Lanes: (1) molecular weight markers, (2) Mix of the PapMV nano, SrtA and the N protein before the coupling reaction, (3) protein profile of the proteins after the coupling reaction. The PapMV-N fusion protein (PapMV CP-N), the free N protein, the SrtA and the PapMV CP are labelled on the right of lane 3. (**C**) Dynamic light scattering (DLS) of the PapMV nano (black line) and the PapMV-N vaccine (blue line). The curves corresponding to each nanoparticle are labelled on the graph.

We have previously demonstrated that coupling vaccine antigens to the surface of PapMV nanoparticles significantly enhances the immune response against the target antigen [17,18]. Consequently, a conjugation strategy using the bacterial transpeptidase Sortase A (SrtA) was employed (Figure 1A). This enzyme catalyzes a stable covalent bond by recognizing the LPETGG donor motif at the C-terminus of the N protein and a poly-glycine acceptor motif on the PapMV nanoparticle surface [17,18,29]. This process results in nanoparticles decorated with the N protein, hereafter referred to as PapMV-N (Figure 1B).

Several coupling conditions were evaluated to identify the optimal parameters meeting the following criteria:

1. The reaction mixture remained clear (no visible precipitation) after conjugation.
2. The conjugated product remained stable for at least five days at 4 °C.
3. The nanoparticles maintained their characteristic flexuous rod-shaped morphology.

**Figure A2:**
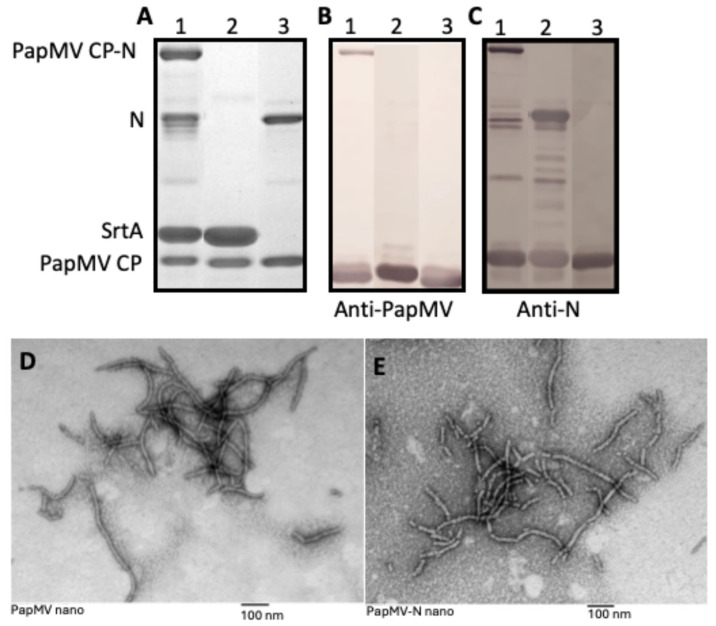
Coupling reaction between SARS-CoV-2 N protein and the PapMV nano. (**A**) SDS-PAGE analysis of the coupling reaction on a Tris-Glycine 10% gel. (**B**) Western Blot using an anti-PapMV polyclonal rabbit antibody. (**C**) Western Blot using an anti-SARS-CoV-2 N rabbit polyconal antibody. Lane 1: Coupling reaction with PapMV nano, SrtA and the N protein; lane 2: Mix of PapMV nano and SrtA; lane 3: Mix of PapMV nano and N protein; lane 4: Mix of SrtA and N protein. (**D**) Electron microscopy imaging of the PapMV nano and (**E**) the PapMV-N nano (right picture). Bars represent 100 nm. Note: The anti-N antibody also recognizes the PapMV CP. It is likely that the 6xH tag used for the purification of these proteins is involved in this cross-reaction.

We determined that a coupling efficiency of 15% for the N protein onto the PapMV coat protein (CP), resulting in the fusion protein PapMV CP-N, best satisfied the three criteria and was therefore selected, as shown in the SDS-PAGE gel (Figure 1B, lane 3). Coupling reactions with efficiencies exceeding 20% tended to induce aggregation, resulting in a cloudy solution. PapMV CP-N consist of the PapMV CP (24.2 kDa) linked to the N protein (47 kDa), which was confirmed by the appearance of a ~71 kDa fusion protein band on SDS-PAGE (Figure 1B, lane 3). Immunoblotting further verified the presence of both the PapMV CP (Figure A2B) and the N protein (Figure A2C) in the PapMV CP-N conjugate. The cross-reaction of the anti-N antibody with PapMV CP is probably due to the recognition of the 6xH purification tag present of both proteins (Figure A2C).

The mean length of the PapMV-N nanoparticles was measured using dynamic light scattering (DLS) and compared to unmodified PapMV nanoparticles (Figure 1C). The PapMV-N particles (114 nm) were slightly larger than the unmodified PapMV particles (81 nm), though both remained within a comparable size range. This modest increase is attributable to the decoration of the nanoparticle surface with the N protein (Figure 1C). The polydispersity index (PDI) of the unmodified PapMV nanoparticles was 0.13, indicating a monodisperse (uniform) population. In contrast, the PDI of the PapMV-N vaccine candidate reached 0.59. While this reflects moderate polydispersity, it remains below the commonly accepted threshold of 0.7, beyond which DLS data are typically considered unreliable. The increased polydispersity was further evidenced by the appearance of two minor secondary peaks in the PapMV-N intensity distribution (accounting for 22% and 6% of the total intensity, respectively), while the primary peak represented 72% of the signal.

Despite this moderate polydispersity, the PapMV-N formulation remained visually clear throughout a 5-day storage period at 4 °C. Consequently, this formulation was selected for further evaluation of its protective potential. Transmission electron microscopy (TEM) confirmed the expected rod-like morphology for both PapMV-N and unmodified PapMV nanoparticles (Figure A2D).

### 3.3. Assessment of the Humoral Response Induced by the N-Based Vaccine Formulations

The recombinant N protein antigen was formulated with well-characterized adjuvants to compare the PapMV nano-adjuvant with established alternatives and help us to classify it among others. The adjuvants used to prepare the vaccine formulations were: (1) CpG ODN, a TLR9 agonist used in the commercial Hepatitis B vaccine (Heplisav-B, Dynavax) that induces a Th1 response [30,31,32]; (2) Alum, a widely used adjuvant found in vaccines such as Hepatitis A (e.g., Havrix, GSK), Hepatitis B (e.g., Recombivax, Merck), Human Papillomavirus (e.g., Gardasil, Merck), and Meningococcal group B (e.g., Trumenba, Pfizer); and (3) PapMV nano, a TLR7/8 agonist [21] known to elicit a strong immune response against antigens coupled to its surface [18].

In each vaccine dose, we mixed 9 µg of the N protein with the different adjuvants. With the CpG ODN adjuvant, 50 µg per dose of vaccine were used (CpG + N). With alum (Adju-Phos^®^), for each vaccine dose, we mixed 50 µL of alum with the N protein (Alum + N). With the PapMV nano, for each vaccine dose, we generated the coupled vaccine by coupling PapMV nano (118 µg) to 9 µg of the N protein (PapMV-N) using 53 µg of SrtA. The reaction was stopped by adding 10 mM EDTA. For the non-coupled vaccine (PpaMV + N), for each dose, we mixed PapMV nano (118 µg) with 9 µg of N, 10 mM EDTA and added 53 µg of the SrtA just prior the immunization to insure that the formulation contains the same components than the PapMV-N vaccine. The compositions were identical between the PapMV + N and the PapMV-N vaccines, only the coupling to the PapMV nano platform differed. The humoral and IFN-γ T-cell responses against the N protein were assessed in animals immunized with these various formulations. 

**Figure 2:**
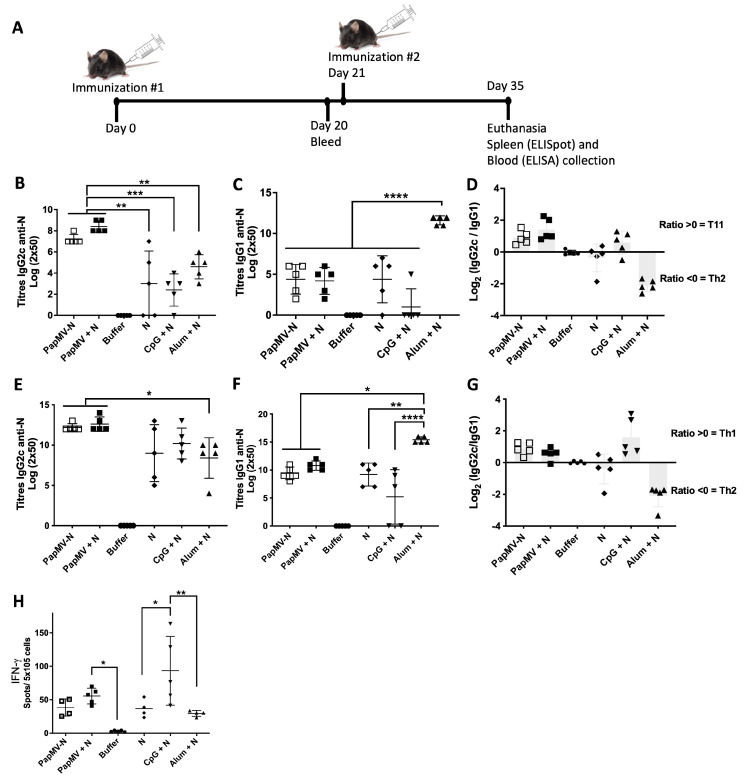
Assessment of the immune response to the N antigen in the different vaccine formulations. (**A**) Schedule of immunizations was created with BioRender (BioRender.com). C57BL/6 mice, 5 per group, were immunized twice, intramuscularly, at day 0 and 21, with the PapMV + N, PapMV-N, CpG + N, Alum + N, N alone vaccines or buffer. Bleeding occurred at day 0, 20 and 40. (**B**) At day 20, the IgG2c titers and (**C**) the IgG1 titers to N and the (**D**) Th1/Th2 ratio were assessed and calculated. (**E**) At day 40, the IgG2c titers and (**F**) the IgG1 titers to N and the (**G**) Th1/Th2 ratio were assessed and calculated. (**H**) The T-cell mediated immune response was assessed by ELISPOT assay using N to stimulate the splenocytes. Number of spots corresponding the number of T cells secreting IFN-γ are shown. * *p* < 0.05, ** *p* < 0.01, *** *p* < 0.001, **** *p* < 0.0001.

C57BL/6 mice (*n* = 10 per group) were immunized on days 0 and 21 via the intramuscular (i.m.) route with each vaccine formulation (Figure 2A). The control group received the buffer used for the coupling reaction. Animals were euthanized on day 35. Blood was harvested on days 20 and 35 for ELISA analysis to evaluate the humoral response to N. Spleens were recovered on day 35 to assess the N-antigen-specific T-cell response by ELISpot. 

On day 20, after the first immunization, the IgG2c response against the N protein was comparable between the PapMV + N and PapMV-N groups; both were higher than the other vaccine formulations (Figure 2B). The Alum + N vaccine was the most effective at inducing the IgG1 antibody subtype (Figure 2C). IgG1 titers for the PapMV + N and PapMV-N vaccines were comparable. The log2 ratio of IgG2c/IgG1 [log2(IgG2c/IgG1)] greater than 0 clearly demonstrates that the CpG ODN and PapMV adjuvants are Th1 polarizing (Figure 2D). As expected, the alum adjuvant elicited a ratio < 0, corresponding to a strong Th2 response to the N antigen (Figure 2D).

By day 35, following two immunizations, the same trend was observed. The PapMV adjuvant, as seen in both the PapMV + N and PapMV-N groups, proved equally effective at inducing IgG2c titers against the N antigen; both groups outperformed the Alum + N vaccine. Conversely, IgG1 titers for the Alum + N vaccine were higher than all other formulations (Figure 2F). As shown in Figure 2D, the IgG2c/IgG1 ratio confirmed that PapMV and CpG ODN act as Th1 adjuvants, while Alum acts as a Th2 adjuvant. Notably, the PapMV nano-adjuvant was more effective than CpG ODN at inducing IgG1 titers, suggesting a more balanced Th1/Th2 response. Finally, to assess the T-cell response, splenocytes were stimulated with the N protein antigen for ELISpot analysis (Figure 2H). The data revealed that the CpG + N vaccine was the most effective at inducing IFN-γ secretion, while Alum + N was the least effective. The efficacy of PapMV + N was statistically comparable to that of the CpG + N formulation. 

**Figure A3:**
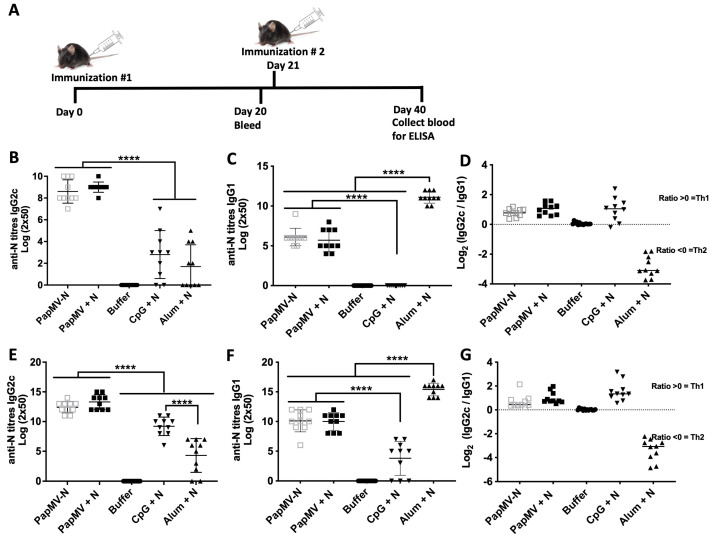
Assessment of the antibody response to the N antigen in the different vaccine formulations. (**A**) Schedule of immunizations was created with BioRender (BioRender.com). C57BL/6 mice, 10 per group, were immunized twice, intramuscularly, at days 0 and 21, with each of the PapMV + N, the PapMV-N, the CpG + N, the Alum + N vaccines or buffer. Bleeding occurred at days 0, 20 and 40. (**B**) At day 20, the IgG2c titers and (**C**) the IgG1 titers to N and the (**D**) Th1/Th2 ratio were assessed and calculated. (**E**) At day 40, the IgG2c titers and (**F**) the IgG1 titers to N and the (**G**) Th1/Th2 ratio were assessed and calculated. **** *p* < 0.0001.

Following a protocol and using the same vaccines as described above, we repeated the experiment with 10 animals per group. We performed two intramuscular (i.m.) immunizations on days 0 and 21, followed by euthanasia on day 40. Blood was harvested on days 20 and 40 (Figure A3). ELISA results confirmed and enhanced the statistical resolution of the data presented in Figure 2. We observed that the CpG + N vaccine was superior to Alum + N in inducing IgG2c production (Figure A3E) after two immunizations. Furthermore, both PapMV + N and PapMV-N were superior to CpG + N and Alum + N in inducing N-protein-specific IgG2c (Figure A3E). The results also confirmed that PapMV-N and PapMV + N vaccines were superior to CpG + N for the induction of IgG1 titers (Figure A3F), while the Alum + N vaccine remained the most effective at inducing IgG1 production against the N protein (Figure A3G).

Unexpectedly, across both experiments, the PapMV + N vaccine was as effective as the PapMV-N vaccine. This finding does not align with previous reports from our group [17,18], which demonstrated that coupling the vaccine antigen to the nanoparticle enhances the immune response compared to the uncoupled formulation. This suggests that the N antigen in the PapMV + N formulation may be interacting with the surface of the PapMV nanoparticle, thereby enhancing the immune response directed toward the antigen.

### 3.4. Assessment of the Binding of N to the PapMV Nano in the PapMV + N Formulation

To investigate if the N protein interacts with the PapMV nano within the PapMV + N formulation, we performed a microscale thermophoresis (MST) experiment using the NanoTemper Monolith NT115 instrument (NanoTemper Technologies GmbH). MST is a fluorescence-based biophysical technique used to quantify the strength of molecular interactions between proteins. This technique relies on the principle that changes in the chemical environment around a fluorophore, when bound to a target molecule that interacts with a ligand, cause a variation in fluorescence intensity that could be interpreted as an interaction between the two molecules.

The PapMV + N vaccines contain the same proteins and buffer composition as the PapMV-N, with the key difference being that SrtA is added to the PapMV + N vaccine, in presence of 10 mm EDTA only prior to animal immunization.

**Figure 3:**
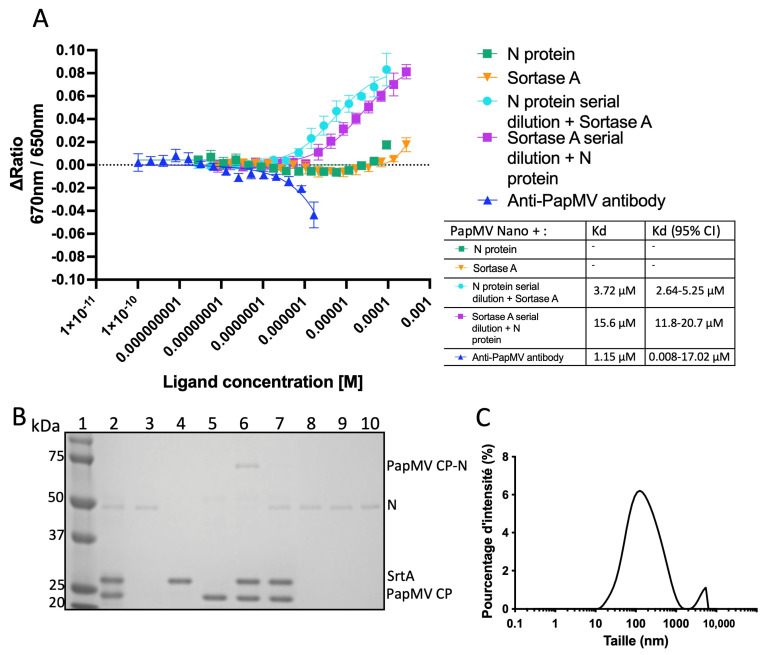
Assessment of the binding of N to the PapMV nano in the PapMV + N vaccine formulation. (**A**) A binding assay using the Monolith instrument allowed measuring the interaction between the PapMV nano, the SrtA and the SARS-CoV-2 N protein. (**B**) SDS-PAGE of the left-over vaccine formulations used to immunize mice of the experiment presented on Figure 2. After immunization, the left-overs were mixed with denaturation dye and snap frozen on dry ice. Lane 1: molecular weight markers. The lanes 2 to 5 freshly prepared and immediately denatured with SDS PAGE loading dye. Lane 2: PapMV nano, N and SrtA; lane 3: N; lane 4: SrtA; lane 5: PapMV nano. The lanes 6 to 10 correspond to the left-overs of the different vaccine formulations. Lane 6: PapMV-N vaccine; lane 7: PapMV + N vaccine; lane 8: PapMV nano + SrtA; lane 9: CpG + N; and lane 10: Alum + N. (**C**) Dynamic light scattering (DLS) of the PapMV + N vaccine formulation containing PapMV nano, the N antigen, the SrtA and EDTA.

For the MST experiment, we labeled the PapMV nano with a fluorescent dye (Cy5). We then introduced the following putative ligands: (1) a monoclonal antibody targeting the PapMV nanoparticle (positive control), (2) the N protein alone, (3) SrtA alone, (4) SrtA with increasing amounts of N protein, and (5) the N protein with increasing amounts of SrtA. The results reveal that in the absence of SrtA, the PapMV nano and the N protein do not interact with each other (Figure 3A-green curve). Similarly, the PapMV nano and SrtA alone also show no interaction (Figure 3A-orange curve). But when the PapMV nano is mixed with both SrtA and increasing amount of the N protein, we observed a measurable interaction with the nanoparticle (Figure 3A-light blue) with a Kd evaluated at 3.72 µM. When increasing amounts of SrtA were added to the PapMV nano and N, we measured a Kd of 15.6 µM (Figure 3A-purple). Finally, for the positive control, the addition of increasing amounts of the monoclonal antibody targeting the PapMV nano showed the strongest binding, with a Kd of 1.15 µM (Figure 3A-dark blue).

Interestingly, the interaction between the PapMV nanoparticle, SrtA, and the N protein occurs even in the presence of 10 mM EDTA, which chelates the Ca^2+^ required for SrtA activity [33].

To verify whether the coupling of PapMV CP and N occurred in the PapMV + N formulation, we analyzed the unused vaccine leftovers post-immunization via SDS-PAGE (Figure 3B). SDS-PAGE denaturation buffer was added to the samples, which were immediately snap-frozen on dry ice. The SDS-PAGE analysis of these samples revealed a band corresponding to the coupled PapMV CP-N in the PapMV-N formulation (Figure 3B, lane 6). Remaining uncoupled N protein was scarcely detectable in this sample, as it had been consumed during ligation with PapMV CP to generate the fusion product (Figure 3B, lane 6). In contrast, no fusion product (PapMV CP-N) was detected in the PapMV + N vaccine formulation (Figure 3B, lane 7). As expected, other vaccine formulations displayed the predicted protein profiles, with only the N protein visible on the gel (Figure 3B, lanes 8–10). Finally, the mean length of the PapMV + N vaccine was measured by DLS (Figure 3C). The PapMV + N formulation showed an average size of 124 nm, which is comparable to that of the 114 nm of the PapMV-N vaccine (see Figure 1C). The PDI of the PapMV + N vaccine was 0.49; while this indicates a moderately polydisperse population, it is notably lower than the PDI of 0.59 observed for the PapMV-N vaccine.

Based on the results presented in Figure 3, we hypothesize that a non-covalent complex forms between the PapMV nanoparticle, SrtA, and the N protein. This complex appears sufficient to enhance the immune response directed toward the N protein, matching the efficacy observed with the PapMV-N vaccine formulation. 

### 3.5. Protection to SARS-CoV-2 Challenges Induced by the PapMV Vaccine Formulations

**Figure 4:**
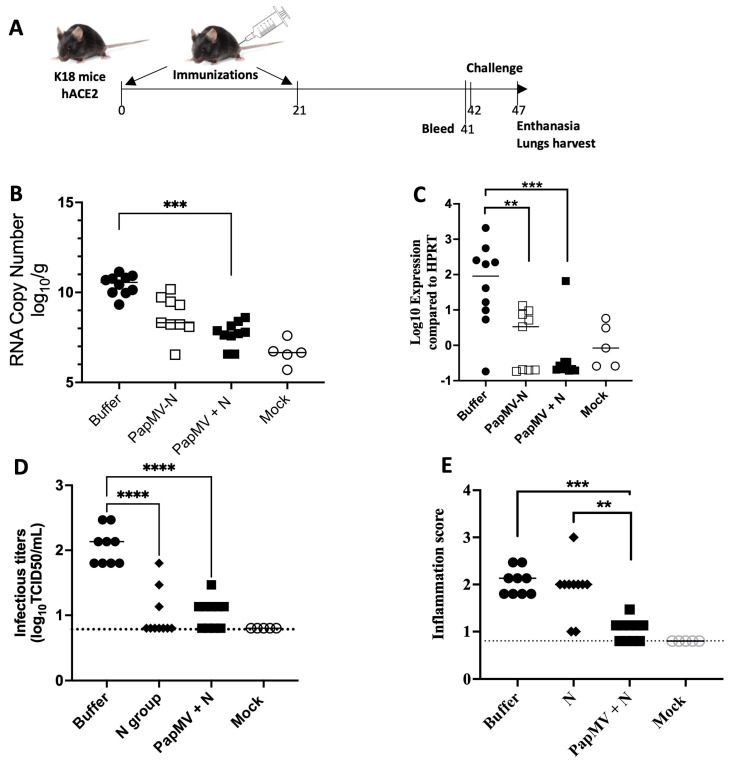
Protection assays of vaccinated mice against SARS-CoV-2 infections. (**A**) Schedule of immunizations was created with BioRender (BioRender.com). K18-hACE2 mice, 10 per group, were vaccinated on days 0 and 21. Blood was collected at day 41 and animals were challenged with SARS-CoV-2 (ancestral variant) on day 42. At day 47 (5 days post-infection), animals were euthanized and the lungs were harvested and homogenized. The viral load (**B**) and the levels of IL-6 (**C**) were assessed by RT-PCR in the lung of infected animals with the ancestral variant of SARS-CoV-2. The viral load, infectious titers assessed in vitro (**D**) and the inflammation score (**E**) were assessed in the lungs of infected animals with the Omicron variant XBB.1.5. The dotted lines correspond to the limit of detection of the infectious titers in (**D**) or the basal level of inflammation score in non-infected animals. ** *p* < 0.01, *** *p* < 0.001, **** *p* < 0.0001.

**Figure A4:**
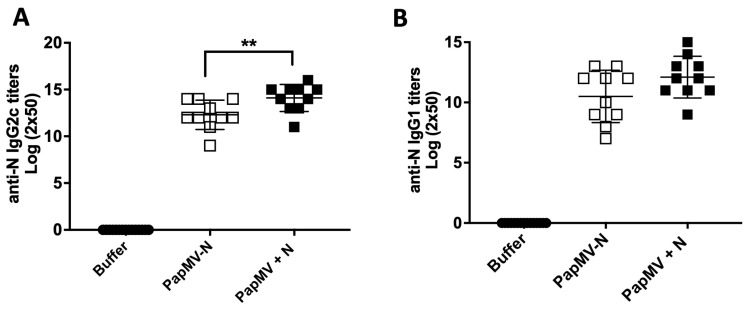
Assessment of the antibody response to the N antigen of the different vaccine formulations prior to the challenge experiment on day 41 with the ancestral variant described in Figure 4A–C. (**A**) IgG2c, and (**B**) IgG1 titers directed to the N protein. ** *p* < 0.01.

To evaluate the capacity of the PapMV nano-based vaccines to elicit protection against a SARS-CoV-2 challenge (ancestral variant), we immunized transgenic mice expressing the human ACE2 receptor (K18-hACE2), which are known to be highly susceptible to SARS-CoV-2 infection [18]. In this experiment, vaccines and controls were administered intramuscularly twice at a 21-day interval (Figure 4A). We immunized 10 mice per group, with three groups receiving either the PapMV-N vaccine, the PapMV + N vaccine, or formulation buffer. A non-vaccinated, non-challenged group of five mice was also included as a control. On day 41, blood was collected to assess the IgG2c titers directed against the N protein (Figure A4). As expected, both the PapMV-N and PapMV + N vaccines were immunogenic. Interestingly, the group receiving the PapMV + N vaccine induced a higher IgG2c antibody response than the PapMV-N coupled group.

On day 42, animals were challenged with the ancestral variant of SARS-CoV-2. Animals were euthanized on day 5 post-challenge, and their lungs were harvested to assess viral titers (Figure 4B) and IL-6 levels (Figure 4C). The weight of the animals were recorded before the challenge and a few hours before the euthanasia and no significant changes in the weight of the animals in all the groups were recorded. Interestingly, viral titers, measured by RT-PCR, were lower in both the PapMV-N and PapMV + N vaccinated groups compared to the buffer control. Additionally, the levels of lung inflammation, which correlate with IL-6 levels [18], were also lower in animals vaccinated with the PapMV-N and PapMV + N. Notably, the PapMV + N vaccinated group showed lower IL-6 levels than the PapMV-N vaccine group, a result likely associated with the superior immunogenicity of the PapMV + N vaccine.

**Figure A5:**
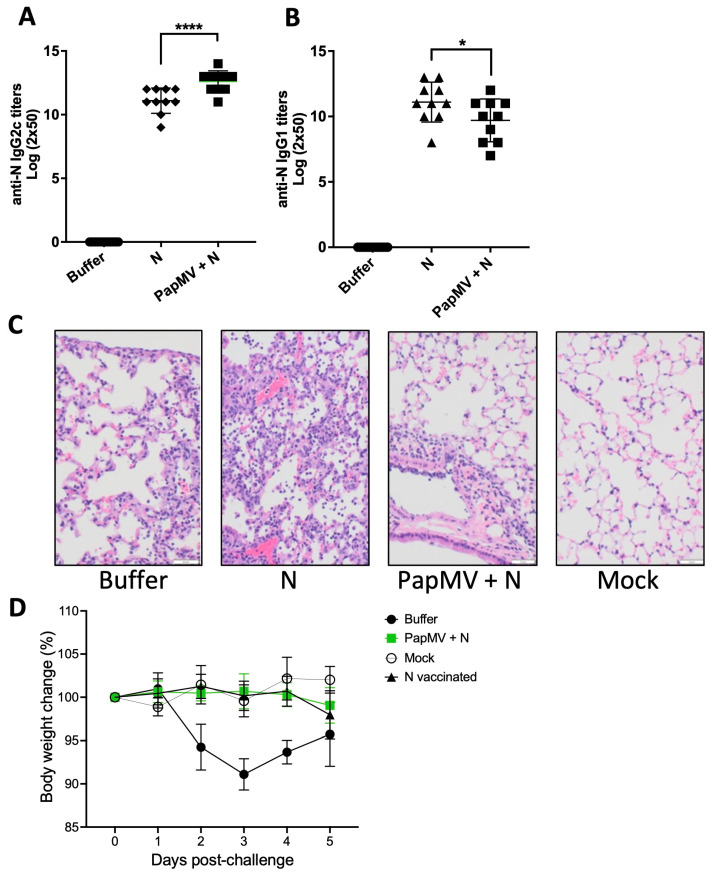
Assessment of the antibody response directed to the N protein, weight losses and IL-6 levels in the challenge experiment described in Figure 4D,E. (**A**) IgG2c, and (**B**) IgG1 titers directed to the N protein at day 41, prior to the challenge with the Omicron XBB.1.5 variant on day 42. (**C**) Immuno-histology of the lungs of infected animals to assess the level of inflammation from day 1 to 5 after infection with the Omicron variant XBB.1.5. (**D**) Weigth loss curve of the animals during 5 days of infection with XBB.1.5. * *p* < 0.05, **** *p* < 0.0001.

To demonstrate the broad protection induced by our leading candidate, the PapMV + N vaccine, we conducted a challenge experiment using the SARS-CoV-2 Omicron XBB.1.5 variant, which is genetically distant from the ancestral strain. Following the established schedule, 10 K18-hACE2 mice per group were immunized with either the formulation buffer, the N protein alone, or the PapMV + N vaccine. A group of five non-vaccinated, non-infected mice served as a mock control. As expected, the PapMV + N vaccine was highly immunogenic and induced significantly higher IgG2c levels than N protein alone (Figure A5A). However, the N-only group showed higher N-specific IgG1 levels compared to the PapMV + N group (Figure A5B), suggesting a stronger bias toward a Th2 response in the absence of the nanoparticle.

Vaccinated animals were challenged with the XBB.1.5 variant on day 42 and euthanized on day 47 to assess pulmonary viral titers (Figure 4D) and inflammation scores (Figure 4E). Both the N and PapMV + N vaccinated groups exhibited reduced viral loads in the lungs compared to the control group (Figure 4D). Notably, the PapMV + N vaccine resulted in lower lung inflammation scores than both the N-only and buffer control groups (Figure 4E and Figure A5C). In contrast with the buffer vaccination control, the PapMV + N vaccinated group did not show significant weight loss during the 5 days of infection with (Figure A5D).

Collectively, these results demonstrate that the PapMV nanoparticle platform, when associated with the N antigen, effectively reduces viral load and lung pathology in mice infected with divergent SARS-CoV-2 variants.

## 4. Discussion

The effectiveness of vaccines depends on their ability to induce neutralizing antibodies, activate cell-mediated immune responses against conserved Cytotoxic T Lymphocyte (CTL) epitopes, or a combination of both [34]. Here, we demonstrated that a vaccine that does not induce neutralizing antibodies, utilizing the SARS-CoV-2 N protein in conjunction with the PapMV nanoparticle platform, was highly immunogenic and reduced viral loads in the lungs of infected animals. Vaccines comprising the PapMV nanoparticle and the N antigen triggered both strong humoral and T-cell immune responses.

After just one immunization, the PapMV-based vaccines (PapMV-N and PapMV + N) elicited a more robust IgG2c response than the commercial adjuvants CpG and Alum. This result aligns with previous studies describing the PapMV nanoparticle as an accelerator of antibody class switching toward higher-affinity antibodies through TLR7 stimulation [18,21,35]. This highlights the clear advantage of using PapMV nanoparticles over other adjuvants, especially during a pandemic, due to their ability to rapidly elicit antigen-specific humoral responses, ensuring faster population protection. Non-neutralizing antibodies targeting the SARS-CoV-2 N protein have been shown by others to confer protection against viral infection [36,37,38,39]. The reduction in severe COVID-19 by N-specific antibodies is also known to be driven by natural killer cell-mediated antibody-dependent cellular cytotoxicity (ADCC) [37,39], which recognizes and kills infected cells by targeting the N protein on their surface [38].

The PapMV-based vaccines (PapMV-N and PapMV + N) also triggered a potent T-cell-mediated immune response to the N antigen. This is consistent with previous findings, which show that attaching a protein antigen to the PapMV nanoparticle surface stimulates the proliferation of antigen-specific T cells in the spleen, leading to IFN-γ secretion upon stimulation [17,18,40,41]. The T-cell response directed to the N antigen likely contributes to the broad immunity elicited by both vaccines.

Unexpectedly, our results show no significant difference in the immune responses between the coupled PapMV-N and the non-coupled PapMV + N vaccine. Our data demonstrate that SrtA creates a bridge between the N protein and the PapMV nanoparticles, leading to the non-covalent attachment of the N antigen to the surface of the nanoparticle. It is likely that this complex improves the antigenic presentation of the N protein to B cells [18] while facilitating the delivery of the N antigen into compartments associated with epitope processing for MHC class I and II presentation, as observed with the coupled PapMV-N vaccine [18,42,43,44]. Both PapMV-N and PapMV + N vaccines reduced viral loads in the lungs following infection with the highly pathogenic ancestral SARS-CoV-2 variant in K18-hACE2 mice. Additionally, we demonstrated that the PapMV + N vaccine also reduced viral load against the Omicron XBB.1.5 variant, highlighting its broad applicability. In both cases, lung inflammation, assessed by IL-6 levels and inflammation scores, was notably lower than in the non-vaccinated group. Furthermore, the PapMV + N vaccine offers a practical advantage over the PapMV-N formulation, which is more prone to aggregation over time. Given that purified SrtA is highly stable in solution, the PapMV + N approach appears to be a more robust option, despite requiring the addition of SrtA prior to immunization.

Previous studies have highlighted negative outcomes for vaccines that elicited an anti-N Th2 immune response [45,46,47,48]. Notably, Deming et al. reported that targeting the N protein as a vaccine antigen failed to protect mice against viral challenge and instead exacerbated the immunopathology of SARS-CoV [46]. They found that mice immunized with Venezuelan equine encephalitis virus replicon particles expressing the N protein experienced adverse outcomes, including eosinophilic infiltration in the lung. Similarly, another study showed that mice immunized with a recombinant vaccinia virus expressing the N protein exhibited severe pneumonia [48]. In both cases, the authors noted that their vaccines induced a Th2-skewed cytokine profile, which likely contributed to the increased immunopathology. It has been proposed that the N protein can act as an interferon antagonist, potentially interfering with the host’s antiviral innate immune response and skewing it toward a Th2-type response [45,49,50].

However, a substantial body of evidence supports the N protein as an excellent target for vaccine development [8,11,51,52,53,54,55,56,57,58]. Many vaccine candidates using the N protein as the primary antigen are highly immunogenic and effectively control SARS-CoV-2 infection in vaccinated animals. Notably, immunization of both K18-hACE2 mice and Syrian hamsters with a human adenovirus type 5 vector (Ad5) expressing only the N protein conferred protection against SARS-CoV-2 by mitigating weight loss and reducing viral loads [52]. In another study, Syrian hamsters immunized with a vaccine co-expressing the membrane (M) and N proteins were protected against severe disease, as evidenced by decreased weight loss, reduced lung pathology, and lower viral titers [56]. Furthermore, in a Rhesus macaque model, animals immunized with a peptide-based vaccine comprising N protein epitopes (adjuvanted with CpG and monophosphoryl lipid A) were moderately protected, showing reduced pneumonia-like damage, fewer cell infiltrates, and lower viral loads [8]. Taken together, these findings support our conclusion that the N protein is a valuable antigen and confirm that its use requires a Th1-orienting adjuvant, such as the PapMV nanoparticle, to induce broad protection. 

In the event of an emerging viral strain, N-protein-based vaccines could serve as an immediate tool to control inflammation, severe disease, and mortality, bridging the gap until strain-specific vaccines become available. Targeting the N protein could also broaden the immune response of S-based vaccines and enhance protection against variants. Indeed, multi-antigenic vaccines targeting both the S and N proteins have demonstrated immunogenicity and protective efficacy against SARS-CoV-2 in animal models [30,59,60,61,62,63,64]. Notably, mRNA vaccines encoding both the N and S proteins enhanced protection against the SARS-CoV-2 Delta and Omicron variants in hamsters, surpassing the effects of mRNA-S alone [60]. Additionally, the combination of Ad5-S and Ad5-N vaccines exhibited synergistic effects, leading to more robust protection against viral invasion of the brain [36].

## 5. Conclusions

Taken together, our findings suggest that combination of the PapMV adjuvant and the N protein generates highly immunogenic vaccines that may be of significant interest as a complement to next-generation vaccines. This study also highlights the critical importance of a Th1-polarized immune response for protection against SARS-CoV-2. Our results support the incorporation of N-based vaccines into global preparedness strategies, marking a significant step forward in our ability to combat future pandemics.

## Data Availability

Data are contained within the article.
